# Survival of African-American and Caucasian men after sipuleucel-T immunotherapy: outcomes from the PROCEED registry

**DOI:** 10.1038/s41391-020-0213-7

**Published:** 2020-02-28

**Authors:** Oliver Sartor, Andrew J. Armstrong, Chiledum Ahaghotu, David G. McLeod, Matthew R. Cooperberg, David F. Penson, Philip W. Kantoff, Nicholas J. Vogelzang, Arif Hussain, Christopher M. Pieczonka, Neal D. Shore, David I. Quinn, Eric J. Small, Elisabeth I. Heath, Ronald F. Tutrone, Paul F. Schellhammer, Matthew Harmon, Nancy N. Chang, Nadeem A. Sheikh, Bruce Brown, Stephen J. Freedland, Celestia S. Higano

**Affiliations:** 1 Tulane Medical School, New Orleans, LA USA; 2 Duke Prostate and Urologic Cancer Center, Duke Cancer Institute, Durham, NC USA; 3 MedStar Southern Maryland Hospital Center, Maryland, MD USA; 4 Center for Prostate Disease Research at the Uniformed Services University of Health Sciences, Bethesda, MD USA; 5 The Walter Reed National Military Medical Center, Bethesda, MD USA; 6 Departments of Urology and Epidemiology and Biostatistics, University of California San Francisco, San Francisco, CA USA; 7 Vanderbilt University Medical Center, Nashville, TN USA; 8 Memorial Sloan Kettering Cancer Center, New York, NY USA; 9 Comprehensive Cancer Centers of Nevada, Las Vegas, NV USA; 10 University of Maryland School of Medicine, Baltimore, MD USA; 11 Associated Medical Professionals, Syracuse, NY USA; 12 Department of Urology, Carolina Urologic Research Center, Myrtle Beach, SC USA; 13 Norris Comprehensive Cancer Center, University of Southern California, Los Angeles, CA USA; 14 UCSF Helen Diller Family Comprehensive Cancer Center, University of California San Francisco, San Francisco, CA USA; 15 Karmanos Cancer Institute, Wayne State University School of Medicine, Detroit, MI USA; 16 Chesapeake Urology Research Associates, Towson, MD USA; 17 Department of Urology, Eastern Virginia Medical School Urology of Virginia, Virginia, VA USA; 18 Dendreon Pharmaceuticals LLC, Seattle, WA USA; 19 Center for Integrated Research in Cancer and Lifestyle, Cedars-Sinai Medical Center, Los Angeles, CA USA; 20 The Durham Veterans Administration, Durham, NC USA; 21 University of Washington and Fred Hutchinson Cancer Research Center, Seattle, WA USA

**Keywords:** Cancer therapy, Cancer therapy, Cancer therapy, Cancer therapy

## Abstract

**Purpose:**

African Americans experience greater prostate cancer risk and mortality than do Caucasians. An analysis of pooled phase III data suggested differences in overall survival (OS) between African American and Caucasian men receiving sipuleucel-T. We explored this in PROCEED (NCT01306890), an FDA-requested registry in over 1900 patients with metastatic castration-resistant prostate cancer (mCRPC) treated with sipuleucel-T.

**Patients and methods:**

OS for patients who received ≥1 sipuleucel-T infusion was compared between African American and Caucasian men using an *all patient* set and a *baseline prostate-specific antigen (PSA)-matched* set (two Caucasians to every one African American with baseline PSAs within 10% of each other). Univariable and multivariable analyses were conducted. Survival data were examined using Kaplan–Meier and Cox proportional hazard methodologies.

**Results:**

Median follow-up was 46.6 months. Overall survival differed between African American and Caucasian men with hazard ratios (HR) of 0.81 (95% confidence interval [CI]: 0.68–0.97, *P* = 0.03) in the *all patient* set and 0.70 (95% CI: 0.57–0.86, *P* < 0.001) in the *PSA-matched* set. Median OS was longer in African Americans than in Caucasian men for both analysis sets, e.g., 35.3 and 25.8 months, respectively, in the *PSA-matched* set. Similar results were observed in the *all patient* set. Differences were larger when treatment began at lower baseline PSA; curves were more similar among patients with higher baseline PSA. In patients with baseline PSA below the median, the HR was 0.52 (95% CI: 0.37–0.72, *P* < 0.001), with median OS of 54.3 versus 33.4 months. Known prognostic factors and African American race (multivariable analyses; HR: 0.60, 95% CI: 0.48–0.74, *P* < 0.001) were independently associated with OS. Use of post-sipuleucel-T anticancer interventions was balanced between races.

**Conclusion:**

In this exploratory analysis of a registry including nearly 12% African American men with mCRPC, OS was significantly different between African Americans and Caucasians, indicating further research is warranted.

## Introduction

Sipuleucel-T, an established autologous cellular immunotherapy for men with asymptomatic/minimally symptomatic metastatic castration-resistant prostate cancer (mCRPC), is recommended as an initial option for treatment by the National Comprehensive Cancer Network Clinical Practice Guidelines in Oncology for Prostate Cancer based on category 1 evidence [1]. Sipuleucel-T improved median overall survival (OS) by 4.1 months versus placebo in the pivotal IMPACT trial (NCT00065442), with a separate post hoc analysis demonstrating a 13-month OS benefit in those with prostate-specific antigen (PSA) levels below the median of 22.1 ng/mL at treatment start [2, 3]. In a contemporary real-world setting of care, data from the PROVENGE Registry for the Observation, Collection, and Evaluation of Experience Data (PROCEED; NCT01306890) found that mCRPC patients treated with sipuleucel-T had a median OS of 30.7 months; with a median follow-up of 46.6 months and an OS of 47.7 months (95% confidence interval [CI], 43.5–50.7 months) in the lowest baseline PSA quartile (≤5.27 ng/mL) [4].

Racial differences in the natural history of prostate cancer and responses to treatments have been described previously; several reviews have discussed different aspects [5–8]. These studies have described differences in biology, access-to-care, socioeconomics, and treatment as well as relative participation in clinical trials [8]. Observed biologic differences include differences within the tumor microenvironment, gene expression, and immunologic pathways [9–14]. Clinically, risk, metastasis rates, and mortality with prostate cancer are higher in African Americans versus Caucasians [15–17].

Yet, several datasets suggest African Americans may have better outcomes with some prostate cancer treatments. A pooled retrospective analysis of eight multicenter trials in “hormone-refractory” prostate cancer showed that black men had significantly lower risk for death than white men [18]. This was recently confirmed by a pooled clinical trial analysis of docetaxel-treated mCRPC men and in a trial of radium-223 [19, 20]. A poster presented at the Genitourinary Symposium in 2019 reported similar observations with enzalutamide and abiraterone acetate [21]. These highlight the importance of understanding race-specific differences to optimize therapy recommendations and the need to overcome the chronic underrepresentation of African American patients in clinical trials.

A retrospective analysis of the clinical trials of sipuleucel-T found a relatively small number of African Americans (*n* = 43, 5.5% of all) enrolled across the three phase III mCRPC studies for sipuleucel-T (including IMPACT [NCT00065442], D9901 [NCT00005947], and D9902A [NCT01133704]). Yet, among these sipuleucel-T-treated African American patients, a 30.7-month OS benefit with sipuleucel-T versus placebo (HR, 0.29; 95% CI, 0.13–0.66, *P* = 0.003) was observed, a primarily hypothesis-generating observation given its post hoc nature and the small number of patients [22–24]. Race was also found to be an independent predictor of OS in the PROCEED registry of sipuleucel-T (HR: 1.64; 95% CI, 1.30–2.06, *P* < 0.001) [4].

To further explore this observed OS benefit, we performed exploratory analyses of data from the PROCEED registry, hypothesizing that African American sipuleucel-T-treated mCRPC patients would have longer OS than their sipuleucel-T-treated Caucasian counterparts.

## Patients and methods

### Patients and treatment

PROCEED was a multicenter, open-label, observational registry in mCRPC patients who received sipuleucel-T, whose primary outcomes have been described previously [4]. Patients were treated at academic and community sites in urology and oncology clinics, from January 27, 2011 to January 17, 2017 [25]. Database lock was May 3, 2017.

Eligible men had to be at least 18 years old with advanced prostate cancer for whom sipuleucel-T treatment was indicated. They could have been enrolled either prospectively before receiving sipuleucel-T or “retrospectively,” defined as “within 6 months after the first leukapheresis.” Race was self-identified as Caucasian, Black or African American, Asian, Native Hawaiian or Other Pacific Islander, or American Indian or Alaska Native.

Sample size (described previously) was chosen to address the primary registry endpoint, which was to assess the cerebrovascular event (CVE) rate [4]. Originally set at 1500 patients it was later increased to 1900 to allow for 4500 person–years of follow-up provided the observed CVE incidence rate was <2.8 per 100 patient–years [4].

Both protocol and amendment were approved by each center’s Institutional Review Board before patient enrollment. Before participation, patients provided written informed consent.

Patients received ≤3 biweekly sipuleucel-T infusions. Sipuleucel-T production has been previously described [26].

### Objectives

The objectives of the analyses presented here are to explore the observations of a difference in survival outcomes between races given the identification of race as an important factor in the multivariate analysis model presented in the primary paper [4], which further describes the outcomes related to the prespecified study objectives of quantifying both the risk of CVEs and survival in all subjects.

### Efficacy

OS was measured from the first sipuleucel-T infusion until date of death as reported by site investigator. Patients were followed for ≥3 years, until death or study withdrawal, or study close. In the few cases where death could not be ascertained, the patient was censored at the date of the last site contact.

Secondarily, the study explored efficacy in terms of race. As part of an exploratory objective, the number and types of OS-prolonging anticancer interventions (ACIs) post-sipuleucel-T were also analyzed.

### Safety

Safety in PROCEED was assessed by recording serious adverse events (SAEs) using MedDRA version 19.1 (for greater details, please see Higano et al.) [4].

### Data analyses

Effect of race on OS in patients treated with sipuleucel-T was assessed by comparing first the subset of all African American and Caucasian men and then the subset of baseline *PSA-matched* African American and Caucasian men. Previously, baseline PSA level was identified as the strongest predictor of post-sipuleucel-T OS [3], and baseline PSA levels were significantly different between African American and Caucasian patients in PROCEED (*P* < 0.001) [4]. Thus, Caucasian subjects were matched (2:1) to African American subjects with baseline PSA levels within ±10% of the Caucasian baseline PSA value to minimize the effect of this clinical imbalance on survival. For these *PSA-matched* cohorts, OS by baseline median PSA levels, PSA quartiles, and post-sipuleucel-T receipt of OS-prolonging ACIs (abiraterone, enzalutamide, docetaxel, cabazitaxel, or radium-223) in mCRPC were assessed using univariable and multivariable analysis (MVA).

OS data were analyzed using Kaplan–Meier methodology, while HRs and 95% CIs were obtained using Cox proportional hazards regression. *P* values were not adjusted for multiplicity as this was an exploratory analysis. Univariable analysis, stepwise Cox modeling, and MVA, with imputation of missing data, were conducted to assess for independent baseline predictors of survival that had both clinical and statistical relevance.

Multiple imputation and statistical analysis were performed using SAS 9.4 and SAS/STAT 14.2 (SAS Institute, Cary NC). In imputing missing data, the Markov Chain Monte Carlo method was used, and all imputations had 15 iterations.

## Results

Median follow-up was 46.6 months. During the follow-up period of >4 years, 1255 patients died; 964 (76.8%) due to prostate cancer progression.

### Patients

Of 1976 patients enrolled, 1902 received ≥1 sipuleucel-T infusion (1649 Caucasians, 221 African Americans, 32 of other race; Supplementary Table S1; disposition, Supplementary Figs. S1, S2). Most patients were treated in oncology practices (1248, 66%), while 654 (34%) patients were treated in urology practices. Most patients (79%) were treated with sipuleucel-T at 140 community clinics, while the remainder were treated at 52 academic centers.

Across all patients, baseline PSA was notably higher in African American patients (33.0 versus 13.9 ng/mL in Caucasians). African Americans exhibited lower hemoglobin, longer time from diagnosis to sipuleucel-T treatment, and lower likelihood of having had prior chemotherapy, regardless of whether looking at the overall population or *PSA-matched* cohorts (Table 1). Two African Americans were excluded in the PSA-matching process: one lacked a baseline PSA, and another had a high PSA value that could not be matched within ±10% to any Caucasian patient (Supplementary Fig. S1).

**Table 1:** Demographics and baseline disease characteristics for the Subset of *PSA-matched* African American and Caucasian patients with mCRPC who were treated with sipuleucel-T in PROCEED.

	Sipuleucel-T-treated, *PSA-matched* patients	
	African American patients (*n* = 219)	Caucasian patients (*n* = 438)
Median (min–max) age, years	71 (42–94)	72 (48–93)
ECOG PS, *n* (%)		
0	138 (63)	299 (68)
1	76 (35)	124 (28)
2	3 (1)	14 (3)
3	1 (0.5)	1 (0.2)
Missing	1 (0.5)	0
Worst Gleason score sum, *n* (%)		
≤6	28 (13)	61 (14)
7	65 (30)	135 (31)
≥8	101 (46)	212 (48)
Missing	25 (11)	30 (7)
Median (Q1–Q3) body weight, kg	88 (79–102)	90 (79–101)
Median (Q1–Q3) PSA, ng/mL	32.9 (8.6–89.7)	28.7 (7.8–82.3)
*n*	219	438
Median (Q1–Q3) LDH, U/L	191 (170–233)	188 (157–220)
*n*	69	165
Median (Q1–Q3) ALP, U/L	88 (69–115)	83 (64–116)
*n*	170	361
Median (Q1–Q3) hemoglobin, g/dL	12.1 (11.0–12.9)	12.9 (11.9–13.7)
*n*	210	417
Median (Q1–Q3) time from diagnosis/biopsy to first infusion, years	5.8 (2.5–10.8)	4.9 (2.5–9.7)
*n*	169	395
Localization of disease, *n* (%)		
Bone only	137 (63)	270 (62)
Bone and lymph nodes	37 (17)	85 (19)
Bone and visceral	5 (2)	8 (2)
Bone, lymph node and visceral	4 (2)	7 (2)
Lymph node only	31 (14)	57 (13)
Lymph node and visceral	2 (1)	1 (0.2)
Visceral	2 (1)	6 (1)
Missing	1 (0.5)	4 (1)
Number of bone metastases, *n* (%)		
*n*	183	370
≤10	129 (71)	277 (75)
>10	27 (15)	76 (21)
Missing	26 (14)	17 (5)
Prior treatment, *n* (%)		
Primary radiation therapy	99 (45)	213 (49)
Radical prostatectomy	69 (32)	148 (34)
Chemotherapy	25 (11)	97 (22)

See Supplementary Table S1 for the comparable table for all African Americans and Caucasian men.

*ALP* alkaline phosphatase, *ECOG PS* Eastern Cooperative Oncology Group performance status, *LDH* lactate dehydrogenase, *mCRPC* metastatic castration-resistant prostate cancer, *PSA* prostate-specific antigen, *Q1* first quartile, *Q3* third quartile.

### Overall survival

**Fig. 1:**
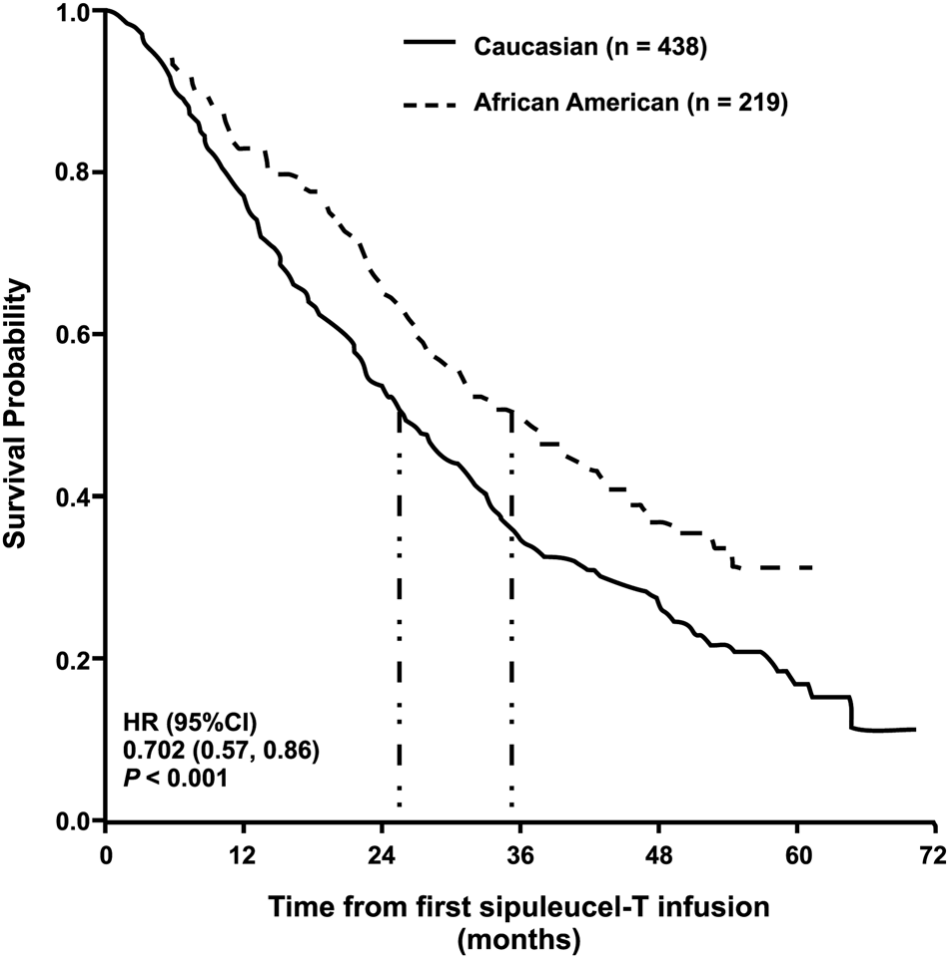
OS in the subset of *PSA-matched* African American and Caucasian men with mCRPC treated with sipuleucel-T in PROCEED for all included patients. Median OS estimates indicated by vertical dash-dot lines. CI confidence interval, HR hazard ratio, OS overall survival, PSA prostate-specific antigen.

Overall survival differed between African American and Caucasian men with a HR of 0.70 (95% CI: 0.57–0.86, *P* < 0.001) in the *PSA-matched* set and 0.81 (95% CI: 0.68–0.97, *P* = 0.03) in the *all patient* set (Fig. 1, Supplemental Figures S1 and S2). Median OS was longer in African Americans than in Caucasian men for both analysis sets (Fig. 1, Supplementary Figs. S1, S2, Table 2). In the *PSA-matched* set, median OS was 35.3 and 25.8 months, respectively (Table 2). The results for the *all patient* set were similar: 35.2 and 29.9 months, respectively.

**Table 2:** Summary of statistical analyses of OS in the subset of *PSA-matched* African American and Caucasian patients with mCRPC who were treated with sipuleucel-T in PROCEED.

Comparison	Median OS, months for sipuleucel-T-treated, *PSA-matched* patients HR (95% CI)^a^		Difference in OS, months	HR (95% CI)^b,c^	*P* value^c^
	African American	Caucasian			
All patients	35.3 (28.7–42.7) *n* = 219^d^	25.8 (22.6–29.0) *n* = 438	9.5	0.70 (0.57–0.86)	<0.001
By median baseline PSA					
≤29.48 ng/mL	54.3 (43.0–NE) *n* = 107	33.4 (29.8–36.6) *n* = 222	20.9	0.52 (0.37–0.72)	<0.001
>29.48 ng/mL	22.7 (19.2–27.6) *n* = 112	17.6 (14.3–22.1) *n* = 216	5.1	0.86 (0.66–1.11)	0.249
By baseline PSA quartile^d^					
≤8 ng/mL	54.3 (43.0–NE) *n* = 53	37.4 (31.6–47.7) *n* = 112	16.9	0.49 (0.29–0.81)	0.005
>8 to ≤29.48 ng/mL	48.8 (31.2–NE) *n* = 54	30.9 (25.0–33.4) *n* = 110	17.9	0.54 (0.35–0.85)	0.008
>29.48 to ≤82.4 ng/mL	27.2 (22.4–33.0) *n* = 56	22.1 (17.5–25.4) *n* = 108	5.1	0.80 (0.55–1.16)	0.236
>82.4 ng/mL	16.4 (10.7–25.1) *n* = 56	13.0 (10.7–18.3) *n* = 108	3.4	0.92 (0.64–1.32)	0.638

Almost all patients who died or left the study did so before the estimated median time in months of 54.3 months. In the group with baseline PSA ≤8 ng/mL, 18 of the 19 patients (who experienced a death) died as of the estimated median survival time of 54.3 months. In the group with baseline PSA > 8 to ≤ 29.48 ng/mL, 24 of the 25 died as of the estimated median survival time of 48.8 months.

*CI* confidence interval, *HR* hazard ratio, *NE* not evaluable, *OS* overall survival, *PSA* prostate-specific antigen.

^a^Kaplan–Meier method.

^b^HR < 1 indicates a better outcome in African American patients treated with sipuleucel-T versus sipuleucel-T-treated *PSA-matched* Caucasians.

^c^A Cox regression model with Caucasians as the reference arm.

^d^PROCEED enrolled 221 African Americans: two were excluded as one patient had no baseline PSA and the other had a baseline PSA of 1462 ng/mL with no matching Caucasian patients.

Baseline PSA impacted survival outcomes, with men with lower baseline PSA exhibiting longer OS (Table 2). Differences between African Americans and Caucasians were statistically significant at lower baseline PSAs, but not at higher baseline PSAs (Table 2); for example, among men with baseline PSA less than the population median, the HR was 0.52 (95% CI, 0.37–0.72, *P* < 0.001) in the *PSA-matched* set.

Univariable analyses (Supplementary Table S2) within the *PSA-matched* set of sipuleucel-T-treated African Americans and Caucasians demonstrated that 12 of the 18 evaluated baseline characteristics were statistically significant in their association with OS, including age, body weight, ethnicity, Eastern Cooperative Oncology Group performance status, PSA, alkaline phosphatase, hemoglobin, lactate dehydrogenase (LDH), lymph node only metastases, prior prostatectomy, prior abiraterone/enzalutamide, and prior docetaxel/cabazitaxel. All but LDH were included in the final primary MVA. LDH was excluded because the amount of missing data was too extensive for data imputation. The resultant final primary multivariable analysis demonstrated that, after adjusting for confounders, African American race is an independent predictor of median OS after sipuleucel-T (Table 3).

**Table 3:** Final primary MVA of OS in the subset of *PSA-matched* African American and Caucasian men with mCRPC treated with sipuleucel-T in PROCEED (*n* = 657).

Baseline covariate	HR (95% CI)	*P* value^a^
Race: African American versus Caucasian	0.60 (0.48–0.74)	<0.001
Age: >median versus ≤ median	1.26 (1.03–1.53)	0.023
Body weight: >median versus ≤ median	0.97 (0.80–1.17)	0.723
ECOG PS: >0 versus 0	1.33 (1.09–1.61)	0.005
Baseline PSA: >median versus ≤ median	1.74 (1.43–2.12)	<0.001
Baseline ALP: >median versus ≤ median	1.59 (1.27–1.99)	<0.001
Baseline hemoglobin: >median versus ≤ median	0.66 (0.54–0.81)	<0.001
Lymph node only metastases: yes versus no	0.66 (0.49–0.90)	0.009
Prior prostatectomy: yes versus no	0.82 (0.68–1.01)	0.058
Prior abiraterone/enzalutamide: yes versus no	1.64 (1.19–2.25)	0.002
Prior docetaxel/cabazitaxel: yes versus no	1.42 (1.11–1.81)	0.005

*ALP* alkaline phosphatase, *CI* confidence interval, *ECOG PS* Eastern Cooperative Oncology Group performance status, *HR* hazard ratio, *MVA* multivariable analysis, *OS* overall survival, *PSA* prostate-specific antigen.

^a^Variables that were statistically significant on univariable analyses were assessed for clinical relevance and included in the final primary multivariable model if deemed both statistically and clinically significant. For multivariable Cox modeling, the Markov chain Monte Carlo imputation method was used for imputing missing data. Parameters with missing data include ECOG PS, ALP, hemoglobin, weight, prior local therapy, and lymph node only metastases. An MVA of the entire PROCEED population was not undertaken due to a major imbalance between the numbers of African American and Caucasian patients, with concern for an underpowered analysis.

In the sensitivity analysis, all ten variables that met the threshold of *P* < 0.1 and had been included in the final stepwise multivariable model (Supplementary Table S3) were also part of the final primary MVA. One difference between the two models was that baseline body weight was excluded from the sensitivity analysis. After adjusting for confounders, African American race was again an independent predictor of OS after sipuleucel-T in this sensitivity analysis.

### Post-sipuleucel-T anticancer intervention

The most commonly used survival-prolonging ACIs after sipuleucel-T were abiraterone, enzalutamide, and docetaxel for both races (Table 4). While all patients were diagnosed with mCRPC, African Americans were more likely to receive hormonal agents, while Caucasians were slightly more likely to be treated with chemotherapy (53% African Americans and 66% Caucasians received chemotherapy). The number of ACIs post-sipuleucel-T was balanced between the *PSA-matched* populations (Table 4).

**Table 4:** Post-sipuleucel-T ACI use in the subset of *PSA-matched* African American and Caucasian men with mCRPC treated with sipuleucel-T in PROCEED.

	Post-sipuleucel-T ACI use in sipuleucel-T-treated, PSA patients	
	African American patients (*n* = 219)	Caucasian patients (*n* = 438)
Number of post-treatment ACIs from five life-prolonging interventions in mCRPC^a^ *n* (%)		
0	45 (21)	113 (26)
1	63 (29)	99 (23)
2	59 (27)	100 (23)
3	36 (16)	80 (18)
4	16 (7)	41 (9)
5	0	5 (1)
Mean number of ACIs (SD)	1.6 (1.2)	1.7 (1.3)
Median number of ACIs (range)	2.0 (0–4)	2.0 (0–5)
Specific post-treatment ACI, *n* (%)		
Abiraterone	123 (56)	234 (53)
Enzalutamide	110 (50)	184 (42)
Docetaxel	80 (37)	194 (44)
Cabazitaxel	36 (16)	96 (22)
Radium-223	4 (2)	20 (5)

*ACI* anticancer intervention, *mCRPC* metastatic castration-resistant prostate cancer, *PSA* prostate-specific antigen, *SD* standard deviation.

^a^ACIs were abiraterone, enzalutamide, docetaxel, cabazitaxel, or radium-223.

### Safety

SAEs occurred in 46 (21%) African Americans treated with sipuleucel-T and 64 (14.6%) *PSA-matched* sipuleucel-T-treated Caucasians. Grade 3–5 SAEs occurred in 32 (15%) African Americans and 48 (11%) *PSA-matched* Caucasians. The incidence of all-grade and grade 3–5 SAEs was low and generally similar between races (Supplementary Table S4).

## Discussion

In PROCEED, the largest, real-world registry with sipuleucel-T in men with mCRPC, the percentage of enrolled African Americans was considerably higher than found in typical advanced prostate cancer trials, allowing for these exploratory analyses of the impact of race on survival to be performed. Overall survival was statistically different between African American and Caucasian men, both among the entire cohort and when examined as a *PSA-matched* set to correct for differences in baseline PSA between the two races (Table 2, Fig. 1). Further, median OS was longer in African Americans than in Caucasian men, regardless of analysis set (Table 2). Detailed multivariable and sensitivity analyses confirmed African American race as an independent predictor of OS with sipuleucel-T, as observed previously [4]. Importantly, variables predictive of OS identified in these analyses were consistent with prior studies.

The OS difference between African Americans and Caucasians treated with sipuleucel-T in PROCEED was more pronounced in mCRPC patients with lower baseline PSA, translating into nearly 4.5 years of survival in the lowest PSA quartile (Table 2). These real-world findings are concordant with observations from the IMPACT study [3]. SAEs did not differ between groups, and no new safety signal emerged from this real-world registry. Together, these analyses support previous findings with sipuleucel-T treatment, where while both groups exhibit a survival benefit, African American men exhibited a greater survival benefit compared with Caucasian men [22], yet it also raises questions as to the reasons why.

Reasons behind the larger representation of African Americans in the current study are unknown, given historically African Americans have been under-represented in clinical trials [8, 27]. One contributing factor may be the time frame of the PROCEED registry (first subject first visit 27 January 2011 and last subject last visit 17 January 2017), a period of significant change in advanced prostate cancer treatment.

The clinical relevance and importance of these findings are highlighted by the fact that prostate cancer is more prevalent in African Americans and more likely to be advanced at presentation, with a greater propensity for progression and lethality, compared to Caucasians [16, 17]. Yet, the underrepresentation of African Americans in clinical trials means there is a paucity of data regarding mCRPC presentation in these two races [8].

Anecdotal evidence suggests differences in the natural history of prostate cancer and in responses to treatment in African American men compared with Caucasian men [5–17]. There are multiple reports of survival benefits with various treatments for mCRPC favoring African American men over Caucasian men [18, 19]. Prior data suggest that African American men with mCRPC have lower risk for death versus Caucasians [18]. Halabi et al. [19] also examined pooled data from nine phase III trials with docetaxel/prednisone in patients with mCRPC in multivariable analysis adjusted for established risk factors; they observed both differences in multiple baseline patient characteristics and a survival benefit (HR: 0.81; 95% CI, 0.72–0.91) favoring African Americans (*n* = 500) versus Caucasians (*n* = 7528) [19]. This was recently confirmed in a trial of radium-223 [20]. McNamara et al. also reported better OS in response to abiraterone acetate or enzalutamide in chemotherapy naïve African American mCRPC patients [21]. These observations align with those found in the current analyses with HRs of 0.70 (95% CI: 0.57–0.86, *P* < 0.001) in the *PSA-matched* set and 0.81 (95% CI: 0.68–0.97, *P* = 0.03) in the *all patient* set (Table 2). Together, these data suggest that African American and Caucasian men with mCRPC may respond differently to systemic therapy with docetaxel, androgen axis inhibitors, or immunotherapy.

Possible mechanisms for greater OS benefit with sipuleucel-T in African Americans are not well understood. One possibility that cannot be confirmed may be that the African American population in PROCEED had relatively more indolent tumor biology, given the longer time to sipuleucel-T therapy initiation. An unlikely possibility for the racial differences in OS observed in PROCEED is the use of subsequent life-prolonging therapies as there was no racial difference in the overall number of survival-prolonging ACIs. Likewise, rates of prior curative local therapy were similar. Use of chemotherapy before sipuleucel-T was higher in Caucasians, despite shorter time from diagnosis to sipuleucel-T treatment. These and other baseline prognostic factors were included in detailed multivariable and sensitivity analyses that found African American race to be independently associated with OS after sipuleucel-T, regardless if looking at the *PSA matched* cohorts or the *all patient* cohorts. Residual confounding effects by unmeasured factors (e.g., inflammation, molecular genotypes, number of circulating tumor cells, diet, and other host factors) may account for the OS difference and should be considered in future studies.

Sipuleucel-T is designed to activate the immune system against the prostatic acid phosphatase expressed on most prostate cancer cells. Sipuleucel-T-induced antigen-specific cellular and humoral immune responses correlate with OS [28, 29]. Thus, immunological differences could be one factor leading to greater OS benefit for African Americans with an immunotherapy. Samples to evaluate peripheral immune responses were not collected in PROCEED, preventing confirmation of the potential role of such immune parameters in mediating the observed differences in OS. Consequently, a hypothesis regarding the role of the immune system in mediating the observed OS differences can only be surmised from published literature.

The existing literature appears to support such a hypothesis of racial differences in the immune system and immune responses. Compared with Caucasians, African Americans, including prostate cancer patients [30], have lower neutrophil and monocyte counts and higher lymphocyte counts [31]. Furthermore, genetic differences in white blood cells, related to cellular immune function, have been reported between African Americans and other races [32]. Differences in gene expression also have been observed in immune response pathways in African American men with aggressive prostate cancer [10, 16, 17]. Racial differences also occur in B-cell and T-cell signaling [33, 34]. Moreover, clinical responses and outcomes vary, e.g., greater vaccine-induced humoral immune responses [35] and higher rejection of organ transplants [36] are observed in African Americans than Caucasians, suggesting more robust immune functionality in the former. Thus, it is plausible that an immunotherapy approach, as with sipuleucel-T, may result in greater immune activation in African American men with mCRPC than in Caucasian men. It, however, should be noted that to date the responsiveness of African Americans to other forms of immune therapy is sparsely documented.

Cancer cells in African American men may be more immunogenic. Tumor mutational burden has been compared in African American men with prostate cancer versus Europeans, suggesting that men of African American ethnicity may have more neoepitopes, plausibly leading to more robust antitumor responses versus Caucasians [37]. Future studies that interrogate the role of the immune response in determining clinical outcome after sipuleucel-T are warranted.

In summary, several reports in the literature describe differences between African Americans and Caucasians in both immunology and cancer biology. Taken together, the clinical outcomes observed with sipuleucel-T may in part be due to such differences, including the immune system’s innate responsiveness and the effectiveness of immunotherapy at the level of the tumor microenvironment. Additional research may help elucidate some of these aspects further.

There are several limitations of the current analyses. PROCEED did not include a comparison arm and the analyses performed were exploratory in nature, so the survival differences observed with sipuleucel-T cannot be confirmed in any of the patients. As this was a registry, race was self-reported without ancestral genotyping. Nevertheless, the strengths of the current analysis include the large numbers in the African American cohort, and the consistency in findings of OS improvement among African Americans compared with Caucasians in both the overall and *PSA-matched* cohorts. African American race also emerged as an independent and strong predictor of OS on multivariable and sensitivity analyses. These data are consistent with the observations of a prior pooled phase III analysis [22]. These observations should motivate new research into immunotherapy responsiveness of African Americans with prostate cancer, with potentially broader implications regarding outcomes among African Americans with other cancers.

## Conclusion

In conclusion, while sipuleucel-T is FDA-approved for men of any race with mCRPC, the PROCEED data suggest that African American men exhibit survival differences compared with Caucasian men when treated with sipuleucel-T, especially at lower PSAs values. Further research is required before this observation is used to drive any potential differential treatment. Rather, the data enhance previous observations as to the utility of sipuleucel-T, including in African American patients with mCRPC. Further research is required to better understand the biologic basis for some of the observed differences between African American and Caucasian men in terms of their responses to immunotherapies and other cancer treatments.

## Supplementary information

Supplementary Material

## References

[1] National Comprehensive Cancer Network. NCCN Clinical Practice Guidelines in Prostate Cancer: version 2.2019, April 17, 2019. National Comprehensive Cancer Network, 2019, pp. NCCN Clinical Practice Guidelines in Oncology (NCCN Guidelines^®^) Prostate Cancer (Version 2.2019, April 2017, 2019).

[2] Kantoff PW, Higano CS, Shore ND, Berger ER, Small EJ, Penson DF (2010). Sipuleucel-T immunotherapy for castration-resistant prostate cancer. N Engl J Med.

[3] Schellhammer PF, Chodak G, Whitmore JB, Sims R, Frohlich MW, Kantoff PW (2013). Lower baseline prostate-specific antigen is associated with a greater overall survival benefit from sipuleucel-T in the Immunotherapy for Prostate Adenocarcinoma Treatment (IMPACT) trial. Urology.

[4] Higano CS, Armstrong AJ, Sartor AO, Vogelzang NJ, Kantoff PW, McLeod DG (2019). Real-world outcomes of sipuleucel-T treatment in PROCEED, a prospective registry of men with metastatic castration-resistant prostate cancer. Cancer.

[5] Smith ZL, Eggener SE, Murphy AB (2017). African-American prostate cancer disparities. Curr Urol Rep.

[6] Bhardwaj A, Srivastava SK, Khan MA, Prajapati VK, Singh S, Carter JE (2017). Racial disparities in prostate cancer: a molecular perspective. Front Biosci (Landmark Ed).

[7] Chornokur G, Dalton K, Borysova ME, Kumar NB (2011). Disparities at presentation, diagnosis, treatment, and survival in African American men, affected by prostate cancer. Prostate.

[8] Ahaghotu C, Tyler R, Sartor O (2016). African American participation in oncology clinical trials–focus on prostate cancer: implications, barriers, and potential solutions. Clin Genitourin Cancer.

[9] Wallace TA, Prueitt RL, Yi M, Howe TM, Gillespie JW, Yfantis HG (2008). Tumor immunobiological differences in prostate cancer between African-American and European-American men. Cancer Res.

[10] Kinseth MA, Jia Z, Rahmatpanah F, Sawyers A, Sutton M, Wang-Rodriguez J (2014). Expression differences between African American and Caucasian prostate cancer tissue reveals that stroma is the site of aggressive changes. Int J Cancer.

[11] Yamoah K, Johnson MH, Choeurng V, Faisal FA, Yousefi K, Haddad Z (2015). Novel biomarker signature that may predict aggressive disease in African American men with prostate cancer. J Clin Oncol.

[12] Powell IJ, Dyson G, Land S, Ruterbusch J, Bock CH, Lenk S (2013). Genes associated with prostate cancer are differentially expressed in African American and European American men. Cancer Epidemiol Biomark Prev.

[13] Huang FW, Mosquera JM, Garofalo A, Oh C, Baco M, Amin-Mansour A (2017). Exome sequencing of African-American prostate cancer reveals loss-of-function ERF mutations. Cancer Discov.

[14] Wang BD, Ceniccola K, Hwang S, Andrawis R, Horvath A, Freedman JA (2017). Alternative splicing promotes tumour aggressiveness and drug resistance in African American prostate cancer. Nat Comm.

[15] DeSantis CE, Miller KD, Goding Sauer A, Jemal A, Siegel RL (2019). Cancer statistics for African Americans, 2019. CA Cancer J Clin.

[16] Rebbeck TR, Devesa SS, Chang BL, Bunker CH, Cheng I, Cooney K (2013). Global patterns of prostate cancer incidence, aggressiveness, and mortality in men of african descent. Prostate Cancer.

[17] Powell IJ, Bock CH, Ruterbusch JJ, Sakr W (2010). Evidence supports a faster growth rate and/or earlier transformation to clinically significant prostate cancer in black than in White American men, and influences racial progression and mortality disparity. J Urol.

[18] Halabi S, Vogelzang NJ, Ou SS, Kelly WK, Small EJ (2006). Clinical outcomes by age in men with hormone refractory prostate cancer: a pooled analysis of 8 Cancer and Leukemia Group B (CALGB) studies. J Urol.

[19] Halabi S, Dutta S, Tangen CM, Rosenthal M, Petrylak DP, Thompson IM (2018). Overall survival of Black and White men with metastatic castration-resistant prostate cancer treated with docetaxel. J Clin Oncol.

[20] Zhao H, Howard L, De Hoedt A, Terris MK, Amling C, Kane C, et al. Racial discrepancies in overall survival among men treated with radium-223. J Urol. 2019. 10.1097/JU.0000000000000524. 10.1097/JU.0000000000000524 31479407

[21] McNamara MA, George DJ, Ramaswamy K, Lechpammer S, Mardekian J, Schultz NM (2019). Overall survival by race in chemotherapy-naïve metastatic castration-resistant prostate cancer (mCRPC) patients treated with abiraterone acetate or enzalutamide. J Clin Oncol.

[22] McLeod DG, Quinn DI, Cullen J, Whitmore JB (2012). 953 Sipuleucel-T in African-Americans: a subgroup analysis of three phase 3 studies of sipuleucel-T in metastatic castrate-resistant prostate cancer. J Urol.

[23] Higano CS, Schellhammer PF, Small EJ, Burch PA, Nemunaitis J, Yuh L (2009). Integrated data from 2 randomized, double-blind, placebo-controlled, phase 3 trials of active cellular immunotherapy with sipuleucel-T in advanced prostate cancer. Cancer.

[24] Small EJ, Schellhammer PF, Higano CS, Redfern CH, Nemunaitis JJ, Valone FH (2006). Placebo-controlled phase III trial of immunologic therapy with sipuleucel-T (APC8015) in patients with metastatic, asymptomatic hormone refractory prostate cancer. J Clin Oncol.

[25] Higano CS, Armstrong AJ, Sartor AO, Vogelzang NJ, Kantoff PW, McLeod DG (2018). Cerebrovascular event (CVE) outcome and overall survival (OS) in patients (pts) treated with sipuleucel-T (sip-T) for metastatic castration-resistant prostate cancer (mCRPC): results from the PROCEED registry. J Clin Oncol.

[26] Small EJ, Fratesi P, Reese DM, Strang G, Laus R, Peshwa MV (2000). Immunotherapy of hormone-refractory prostate cancer with antigen-loaded dendritic cells. J Clin Oncol.

[27] Spratt DE, Osborne JR (2015). Disparities in castration-resistant prostate cancer trials. J Clin Oncol.

[28] Sheikh NA, Petrylak D, Kantoff PW, Dela Rosa C, Stewart FP, Kuan LY (2013). Sipuleucel-T immune parameters correlate with survival: an analysis of the randomized phase 3 clinical trials in men with castration-resistant prostate cancer. Cancer Immunol Immunother.

[29] Antonarakis ES, Small EJ, Petrylak DP, Quinn DI, Kibel AS, Chang NN (2018). Antigen-specific CD8 lytic phenotype induced by sipuleucel-T in hormone-sensitive or castration-resistant prostate cancer and association with overall survival. Clin Cancer Res.

[30] Vidal AC, Howard LE, de Hoedt A, Cooperberg MR, Kane CJ, Aronson WJ (2018). Neutrophil, lymphocyte and platelet counts, and risk of prostate cancer outcomes in white and black men: results from the SEARCH database. Cancer Causes Control.

[31] Lim EM, Cembrowski G, Cembrowski M, Clarke G (2010). Race‐specific WBC and neutrophil count reference intervals. Int J Lab Hematol.

[32] Keller MF, Reiner AP, Okada Y, van Rooij FJA, Johnson AD, Chen M-H (2014). Trans-ethnic meta-analysis of white blood cell phenotypes. Hum Mol Genet.

[33] Longo DM, Louie B, Mathi K, Pos Z, Wang E, Hawtin RE (2012). Racial differences in B cell receptor signaling pathway activation. J Transl Med.

[34] Sugimoto K, Stadanlick J, Ikeda F, Brensinger C, Furth EE, Alter HJ (2003). Influence of ethnicity in the outcome of hepatitis C virus infection and cellular immune response. Hepatology.

[35] Kurupati R, Kossenkov A, Haut L, Kannan S, Xiang Z, Li Y (2016). Race-related differences in antibody responses to the inactivated influenza vaccine are linked to distinct pre-vaccination gene expression profiles in blood. Oncotarget.

[36] Morris AA, Cole RT, Veledar E, Bellam N, Laskar SR, Smith AL (2013). Influence of race/ethnic differences in pre-transplantation panel reactive antibody on outcomes in heart transplant recipients. J Am Coll Cardiol.

[37] Jaratlerdsiri W, Chan EKF, Gong T, Petersen DC, Kalsbeek AMF, Venter PA (2018). Whole-genome sequencing reveals elevated tumor mutational burden and initiating driver mutations in African men with treatment-naïve, high-risk prostate cancer. Cancer Res.

