# Supplementary material for: Survival of African-American and Caucasian men after sipuleucel-T immunotherapy: outcomes from the PROCEED registry
Source: Prostate Cancer Prostatic Dis. 2020 Feb 28;23(3):517–26. doi: 10.1038/s41391-020-0213-7 (PMC7423504; doi:10.1038/s41391-020-0213-7)
Supplement: Supplementary file 1 — Supplementary Material [file 41391_2020_213_MOESM1_ESM.docx]

Supplementary Material for:

**Survival of African-American and Caucasian Men after Sipuleucel-T Immunotherapy: Outcomes from the PROCEED Registry**

Oliver Sartor^1^*, Andrew J. Armstrong^2^*, Chiledum Ahaghotu^3^, David G. McLeod^4^, Matthew R. Cooperberg^5^, David F. Penson^6^, Philip W. Kantoff^7^, Nicholas J. Vogelzang^8^, Arif Hussain^9^, Christopher M. Pieczonka^10^, Neal D. Shore^11^, David I. Quinn^12^, Eric J. Small^13^, Elisabeth I. Heath^14^, Ronald F. Tutrone^15^, Paul F. Schellhammer^16^, Matthew Harmon^17^, Nancy N. Chang^17^, Nadeem A. Sheikh^17^, Bruce Brown^17^, Stephen J. Freedland^18^**, and Celestia S. Higano^19^**

*Drs Sartor and Armstrong are joint lead authors and equal contributors; **Drs Freedland and Higano are joint senior authors and equal contributors

**Corresponding author**: A. Oliver Sartor, MD, Tulane Medical School, 1430 Tulane Ave, #8642, New Orleans, LA 70112; e-mail: [osartor@tulane.edu](mailto:osartor@tulane.edu)

Tel: +1-504-988-7869; Fax: +1-504-988-1813

**Supplementary Table S1.** Demographics and Baseline Disease Characteristics for all African American and Caucasian Patients with mCRPC Treated with Sipuleucel-T in PROCEED

|  | All Sipuleucel-T-treated Patients | |
| --- | --- | --- |
| Characteristics | African American Patients  (n = 221) | Caucasian Patients  (n = 1 649) |
| Median (range) age, years | 71 (42-94) | 72 (43-97) |
| ECOG PS, n (%) |  |  |
| 0 | 139 (63) | 1 102 (67) |
| 1 | 77 (35) | 487 (30) |
| 2 | 3 (1) | 31 (2) |
| 3 | 1 (0.5) | 7 (0.4) |
| Missing | 1 (0.5) | 22 (1) |
| Worst Gleason score sum, n (%) |  |  |
| ≤ 6 | 29 (13) | 186 (11) |
| 7 | 65 (29) | 500 (30) |
| ≥ 8 | 102 (46) | 843 (51) |
| Missing | 25 (11) | 120 (7) |
| Median (Q1-Q3) body weight, kg | 88 (79-102) | 90 (80-102) |
| Median (Q1-Q3) PSA, ng/mL | 33.0 (8.6-90.4) | 13.9 (5.0-39.6) |
| n | 220 | 1634 |
| Median (Q1-Q3) LDH, U/L | 191 (170-233) | 185 (157-216) |
| n | 69 | 559 |
| Median (Q1-Q3) ALP, U/L | 88 (69-115) | 81 (62-115) |
| n | 170 | 1 303 |
| Median (Q1-Q3) hemoglobin, g/dL | 12.1 (11.0-12.9) | 12.9 (11.9-13.8) |
| n | 212 | 1 552 |
| Median (Q1-Q3) time from diagnosis/biopsy to first infusion, years  n | 6.0 (2.5-10.8)  171 | 5.0 (2.2-9.1)  1 400 |
| Localization of disease, n (%) |  |  |
| Bone only | 138 (62) | 1 066 (65) |
| Lymph node only | 32 (15) | 222 (14) |
| Both bone + lymph node  Visceral | 37 (17)  13 (6) | 270 (16)  73 (4) |
| Missing | 1 (0.5) | 18 (1) |
| Number of bone metastases, n (%) |  |  |
| n | 183 | 1 381 |
| ≤ 10 | 129 (71) | 970 (70) |
| > 10 | 28 (15) | 239 (17) |
| Missing | 26 (14) | 172 (13) |
| Prior treatment, n (%) |  |  |
| Primary radiation therapy | 121 (55) | 821 (50) |
| Radical prostatectomy | 70 (32) | 609 (37) |
| Chemotherapy | 25 (11) | 236 (14) |

Abbreviations: African American, African American; ALP, alkaline phosphatase; ECOG PS, Eastern Cooperative Oncology Group performance status; LDH, lactate dehydrogenase; mCRPC, metastatic castration-resistant prostate cancer; PSA, prostate-specific antigen; Q1, first quartile; Q3, third quartile.

**Supplementary Table S2.** Proportional Hazards Model Assessment of Baseline Predictors of OS in the Subset of PSA-matched African American and Caucasian Men with mCRPC Treated with Sipuleucel-T in PROCEED

|  |  | Univariable Analysis | |
| --- | --- | --- | --- |
| Covariate | n | HR (95% CI) | *P* Value* |
| Age: > median versus ≤ median | 657 | 1.38 (1.15–1.66) | < 0.001 |
| ECOG PS: 1 versus 0 | 656 | 1.48 (1.23–1.79) | < 0.001 |
| Time from diagnosis to sipuleucel-T: > median versus ≤ median^†^ | 564 | 0.85 (0.70–1.04) | 0.111 |
| Baseline PSA: > median versus ≤ median | 657 | 2.10 (1.75–2.53) | < 0.001 |
| Baseline LDH: > median versus ≤ median^‡^ | 234 | 1.37 (1.01–1.84) | 0.040 |
| Baseline ALP: > median versus ≤ median^§^ | 531 | 1.86 (1.52–2.28) | < 0.001 |
| Baseline hemoglobin: > median versus ≤ median^¶^ | 627 | 0.65 (0.53–0.78) | < 0.001 |
| Gleason sum: ≥ 8 versus ≤ 7 | 602 | 1.06 (0.87–1.28) | 0.565 |
| Race: Caucasians versus African Americans | 657 | 1.42 (1.16–1.75) | < 0.001 |
| Body weight: > median versus ≤ median | 656 | 0.77 (0.64–0.93) | 0.006 |
| Practice type: oncology versus urology | 657 | 1.11 (0.90–1.37) | 0.323 |
| Lymph node metastases only: yes versus no^ǁ^ | 652 | 0.59 (0.43–0.79) | < 0.001 |
| Visceral metastases: yes versus no^ǁ^ | 652 | 1.10 (0.73–1.64) | 0.648 |
| Radical prostatectomy: yes versus no | 657 | 0.81 (0.66–0.98) | 0.030 |
| Primary radiation: yes versus no | 657 | 1.09 (0.91–1.31) | 0.348 |
| Radical prostatectomy or primary radiation: yes versus no | 657 | 0.94 (0.77–1.13) | 0.494 |
| Prior docetaxel or cabazitaxel: yes versus no | 657 | 1.59 (1.26 to -2.00) | < 0.001 |
| Prior abiraterone or enzalutamide: yes versus no | 657 | 1.78 (1.32–2.41) | < 0.001 |

Abbreviations: African American, African American; ALP, alkaline phosphatase; CI, confidence interval; ECOG PS, Eastern Cooperative Oncology group performance status; HR, hazard ratio; LDH, lactate dehydrogenase; OS, overall survival; PSA, prostate-specific antigen.

*From a Cox regression model with a single covariate as the independent variable.

^†^Data are missing for 93 patients.

^‡^Data are missing for 423 patients.

^§^Data are missing for 126 patients.

^¶^Data are missing for 30 patients.

^ǁ^Five patients did not have data reported for bone, lymph node or visceral metastases.

Note: This univariable analysis examined baseline characteristics known to be or to be potentially associated with OS in patients with mCRPC and assessed them for statistical significance by generating a series of Cox models, each with a single independent baseline parameter using the ‘complete case’ approach, with no imputations for missing data.

**Supplementary Table S3.** Sensitivity Analysis with Final Stepwise MVA of OS in the Subset of PSA-matched African American and Caucasian Men with mCRPC Treated with Sipuleucel-T in PROCEED (n = 657)

| Baseline Covariate | HR (95% CI) | *P* Value^*^ |
| --- | --- | --- |
| Race: African American versus Caucasian | 0.61 (0.49–0.75) | <0.001 |
| Age: > median versus ≤ median | 1.26 (1.04–1.53) | .017 |
| ECOG PS: > 0 versus 0 | 1.32 (1.09–1.61) | .005 |
| Baseline PSA: > median versus ≤ median | 1.75 (1.44–2.13) | <0.001 |
| Baseline ALP: > median versus ≤ median | 1.53 (1.24–1.89) | <0.001 |
| Baseline hemoglobin: > median versus ≤ median | 0.67 (0.54–0.81) | <0.001 |
| Lymph node only metastases: yes versus no | 0.66 (0.48–0.89) | 0.007 |
| Prior prostatectomy: yes versus no | 0.83 (0.68–1.01) | 0.059 |
| Prior abiraterone/enzalutamide: yes versus no | 1.62 (1.18–2.23) | 0.003 |
| Prior docetaxel/cabazitaxel: yes versus no | 1.41 (1.11–0.81) | 0.006 |

Abbreviations: African American, African American; ALP, alkaline phosphatase; CI, confidence interval; ECOG PS, Eastern Cooperative Oncology Group performance status; HR, hazard ratio; MVA, multivariable analysis; OS, overall survival; PSA, prostate-specific antigen.

* Stepwise Cox modelling; As a sensitivity analysis, stepwise selection was applied to potentially prognostic candidate variables, and variables that met the statistical threshold of *P*<0.1 were included in the final stepwise MVA. Missing data were imputed as described above. Stepwise Cox modelling; 0.10 was used as the enter and stay probabilities for the stepwise selection; the Markov chain Monte Carlo imputation method was used for imputing missing data. Parameters with missing data are: ECOG PS, ALP, hemoglobin, weight, prior local therapy, and lymph node only metastases; parameters considered were median of age, weight, PSA, ALP, hemoglobin, time from diagnosis to treatment, race, ECOG PS, lymph node only, bone and lymph nodes, radical prostatectomy, prior docetaxel/cabazitaxel, prior abiraterone/enzalutamide, and Gleason sum.

**Supplementary Table S4.** Incidence of All-grade SAEs in ≥2 Patients and Grade 3-5 SAEs Among Subsets of PSA-matched African American and Caucasian Men with mCRPC Treated with Sipuleucel-T in PROCEED

|  | SAE*, n (%)  in Sipuleucel-T-treated, PSA-matched Patients | | | | | |
| --- | --- | --- | --- | --- | --- | --- |
|  | African American Patients  (n = 219) | |  | Caucasian Patients  (n = 438) | |  |
|  | All Grades | Grade 3-5 |  | All Grades | Grade 3-5 |  |
| **Any SAE^*^** | 46 (21) | 32 (15) |  | 64 (15) | 48 (11) |  |
| Disease progression | 4 (1.8) | 3 (1.4) |  | 11 (2.5) | 10 (2.3) |  |
| Chills | 1 (0.5) | 0 |  | 5 (1.1) | 0 |  |
| Myocardial infarction | 0 | 0 |  | 5 (1.1) | 4 (0.9) |  |
| Syncope | 1 (0.5) | 1 (0.5) |  | 5 (1.1) | 2 (0.5) |  |
| Anemia | 3 (1.4) | 1 (0.5) |  | 4 (0.9) | 1 (0.2) |  |
| Cerebrovascular accident | 4 (1.8) | 3 (1.4) |  | 4 (0.9) | 4 (0.9) |  |
| Subdural hematoma | 2 (0.9) | 2 (0.9) |  | 4 (0.9) | 4 (0.9) |  |
| Acute kidney injury | 1 (0.5) | 1 (0.5) |  | 3 (0.7) | 3 (0.7) |  |
| Chest pain | 1 (0.5) | 0 |  | 3 (0.7) | 1 (0.2) |  |
| Pyrexia | 2 (0.9) | 0 |  | 3 (0.7) | 1 (0.2) |  |
| Transient ischemic attack | 1 (0.5) | 0 |  | 3 (0.7) | 1 (0.2) |  |
| Urinary tract infection | 1 (0.5) | 0 |  | 3 (0.7) | 2 (0.5) |  |
| Bacteremia | 1 (0.5) | 0 |  | 2 (0.5) | 1 (0.2) |  |
| Blood pressure increased | 0 | 0 |  | 2 (0.5) | 0 |  |
| Cerebral hemorrhage | 2 (0.9) | 2 (0.9) |  | 2 (0.5) | 2 (0.5) |  |
| Confusional state | 0 | 0 |  | 2 (0.5) | 1 (0.2) |  |
| Dehydration | 2 (0.9) | 1 (0.5) |  | 2 (0.5) | 2 (0.5) |  |
| Fall | 0 | 0 |  | 2 (0.5) | 2 (0.5) |  |
| Hypotension | 2 (0.9) | 1 (0.5) |  | 2 (0.5) | 1 (0.2) |  |
| Nausea | 0 | 0 |  | 2 (0.5) | 1 (0.2) |  |
| Pulmonary embolism | 1 (0.5) | 1 (0.5) |  | 2 (0.5) | 2 (0.5) |  |
| Subarachnoid hemorrhage | 0 | 0 |  | 2 (0.5) | 0 |  |
| Thrombosis | 0 | 0 |  | 2 (0.5) | 0 |  |
| Vomiting | 0 | 0 |  | 2 (0.5) | 1 (0.2) |  |
| Dyspnea | 3 (1.4) | 2 (0.9) |  | 1 (0.2) | 1 (0.2) |  |
| Device-related infection | 2 (0.9) | 2 (0.9) |  | 1 (0.2) | 1 (0.2) |  |
| Pneumonia | 2 (0.9) | 1 (0.5) |  | 1 (0.2) | 0 |  |
| Spinal cord compression | 2 (0.9) | 2 (0.9) |  | 1 (0.2) | 1 (0.2) |  |
| Cerebral infarction | 2 (0.9) | 2 (0.9) |  | 0 | 0 |  |

Abbreviations: African American, African American; PSA, prostate-specific antigen; SAE, serious adverse event.

*SAEs are listed in order of decreasing frequency for the Caucasian patients (all-grade SAEs) treated with sipuleucel-T.

**Supplementary Figure S1. ‘CONSORT’ Diagram for the African American Analysis of PROCEED.** PSA, Prostate-specific Antigen.

**
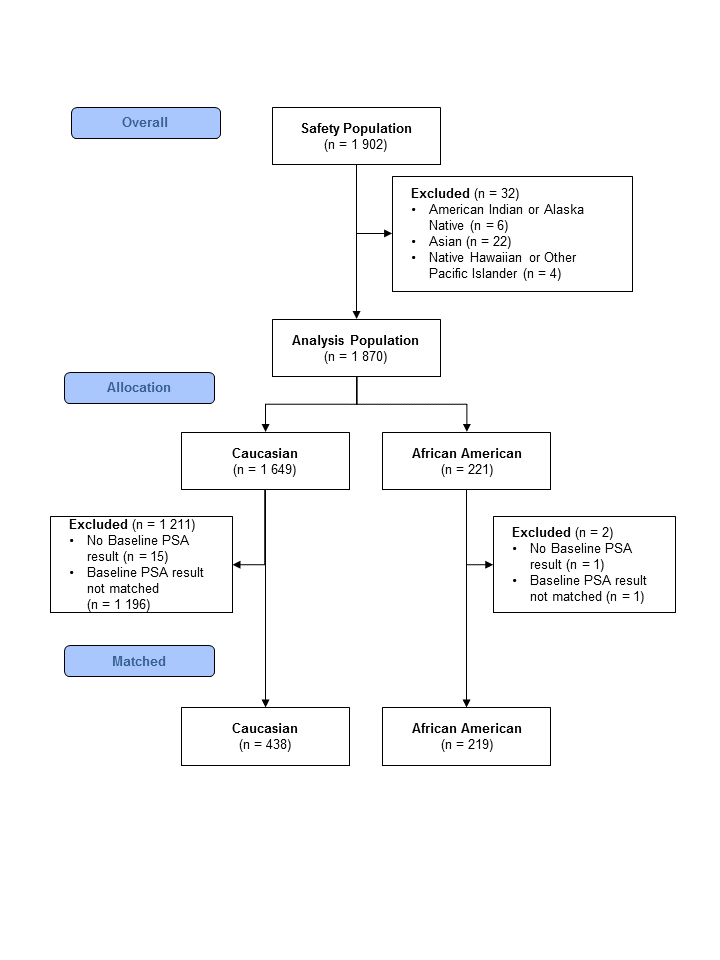
**

**Supplementary Figure S2. Subject Disposition Diagram for PROCEED.** PSA, Prostate-specific Antigen.
